# Immune-related adverse events: a retrospective look into the future of oncology in the intensive care unit

**DOI:** 10.1186/s13613-020-00761-w

**Published:** 2020-10-16

**Authors:** Adrien Joseph, Audrey Simonaggio, Annabelle Stoclin, Antoine Vieillard-Baron, Guillaume Geri, Stéphane Oudard, Jean-Marie Michot, Olivier Lambotte, Elie Azoulay, Virginie Lemiale

**Affiliations:** 1 U1138. INSERM, Équipe 11 labellisée Ligue Nationale Contre Le Cancer, « Metabolism, Cancer & Immunity », Centre de Recherche Des Cordeliers, 15. rue de l’École de Médecine, 75006 Paris, France; 2 Metabolomics and Cell Biology Platforms, Gustave Roussy Cancer Campus, Villejuif, France; 3 Service de Réanimation Médicale, Hôpital Saint-Louis, Assistance Publique Hôpitaux de Paris, Paris, France; 4 Service d’oncologie Médicale, Hôpital Européen Georges Pompidou, Assistance Publique Hôpitaux de Paris, Paris, France; 5 Université de Paris, Paris, France; 6 Service de Médecine Intensive Réanimation, Département Interdisciplinaire de L’Organisation Des Parcours Patient, Institut Gustave Roussy, Villejuif, France; 7 Service de Médecine Intensive Réanimation, Hôpital Ambroise Paré, Assistance Publique Hôpitaux de Paris, Boulogne Billancourt, France; 8 INSERM UMR1018, Team Kidney and Heart, CESP, Villejuif, France; 9 UFR des Sciences de la Santé Simone Veil, Université Versailles Saint Quentin, Versailles, France; 10 Faculté de médecine, Université Paris Saclay, Le Kremlin-Bicêtre, France; 11 Département D’innovations Thérapeutiques Et D’essais Précoces (DITEP), Institut Gustave Roussy, Villejuif, France; 12 Service de Médecine Interne Et D’immunologie Clinique, Hôpital Bicêtre, Assistance Publique Hôpitaux de Paris, 94270 Le Kremlin-Bicêtre, France; 13 Inserm, CEA, Centre de Recherche en Immunologie Des Infections Virales Et Des Maladies Auto-Immunes ImVA, UMR1184, Université Paris-Saclay, 94270 Le Kremlin Bicêtre, France; 14 U1153, INSERM, Équipe ECSTRA, Biostatistiques Et Épidémiologie Clinique, Université de Paris, Paris, France

**Keywords:** Cancer, Outcome, Adverse event, Immunotherapy, Intensive care, Immune-related adverse events, Immune checkpoint inhibitor

## Abstract

**Background:**

Immune checkpoint inhibitors have reshaped the standard of care in oncology. However, they have been associated with potentially life-threatening immune-related adverse events. With the growing indications of immune checkpoint inhibitors and their position as a pillar of cancer treatment, intensive care physicians will be increasingly confronted with their side effects. The outcome of patients with severe immune-related adverse events in the intensive care unit remains unknown. This retrospective multicentric study aims to describe the characteristics of patients admitted to the intensive care units of 4 academic hospitals in Paris area while receiving immune checkpoint inhibitor treatment between January 2013 and October 2019.

**Results:**

Over the study period, 112 cancer patients who received immune checkpoint inhibitors were admitted to the intensive care unit within 60 days after the last dose. ICU admission was related to immune-related adverse events (*n* = 29, 26%), other intercurrent events (*n* = 39, 35%), or complications related to tumor progression (*n* = 44, 39%). Immune-related adverse events were pneumonitis (*n* = 8), colitis (*n* = 4), myocarditis (*n* = 3), metabolic disorders related to diabetes (*n* = 3), hypophysitis (*n* = 2), nephritis (*n* = 2), meningitis or encephalitis (*n* = 2), hepatitis (*n* = 2), anaphylaxis (*n* = 2) and pericarditis (*n* = 1). Primary tumors were mostly melanomas (*n* = 14, 48%), non-small-cell lung cancers (*n* = 7, 24%), and urothelial carcinomas (*n* = 5, 17%). Diagnosis of melanoma and a neutrophil/lymphocyte ratio < 10 were associated with immune-related diagnosis versus other reasons for ICU admission. During their ICU stay, immune-related adverse events patients needed vasopressors (*n* = 7), mechanical ventilation (*n* = 6), and extra-corporeal membrane oxygenation (*n* = 2). One-year survival was significantly higher for patients admitted for irAE compared to patients admitted for other reasons (*p* = 0.004).

**Conclusions:**

Admission to the intensive care unit related to immune-related adverse event was associated with better outcome in cancer patients treated with immune checkpoint inhibitors. Our results support the admission for an intensive care unit trial for patients with suspected immune-related adverse events.

## Background

Immune checkpoint inhibitors (ICI) have revolutionized cancer care and have led to a significant survival improvement in a large variety of tumors [1].

Pharmaceutical specialties currently approved enhance antitumor immunity by reversing tumor escape caused by two negative regulators: *cytotoxic T-lymphocyte antigen 4* (CTLA-4) and *programmed cell death 1* (PD-1) or its ligand, *programmed cell death ligand 1* (PD-L1).

Since the early 2010s, with ICIs coming of age and the tremendous interest they engendered, a new profile of toxicity also revealed itself. Termed *immune related adverse events* (irAEs), the side effects of these novel therapeutic antibodies result from the loss of immune homeostasis and off-target effects in peripheral tissues [2, 3]. Although the skin, endocrine glands and digestive tract are mostly affected, pulmonary [4–6], neurologic [7–10], hepatic [11–13] and cardiologic [14–19] side effects have also been described and may be life-threatening.

The best standard of care for irAEs has not been established through randomized trials and research on the subject is considered an urgent need [6], but guidelines based on experts’ opinion often place steroids as a first-line therapy [20, 21], followed by other immunosuppressive therapies according to the type of irAE and the organ involved [22–25].

With the growing indications of ICI and their position as a pillar of cancer treatment, intensive care physicians will be increasingly confronted with their side effects.

The outcome of patients admitted to the ICU for irAEs remains unknown and may potentially differ from other oncological complications of patients admitted to the ICU [26].

This study aims to describe the characteristics of cancer patients receiving ICI and admitted to the ICU. Patients admitted for irAE were compared to patients admitted for other reasons.

## Methods

We conducted a retrospective multicentric study including patients from 4 French university hospitals in Paris area. All centers were oncologic centers and were organized with a multidisciplinary board [27] to discuss the management of immune-related adverse events. Patients eligible were admitted to ICUs between January 2013 and October 2019, during the course of an ICI treatment (either anti-PD-1 (NIVOLUMAB, PEMBROLIZUMAB or SPARTALIZUMAB), anti-PDL-1 (ATEZOLIZUMAB, DURVALUMAB), anti-CTLA4 (IPILIMUMAB or TREMELIMUMAB) or a combination of ICI.

All consecutive adult patients admitted to the ICU who were receiving an ICI treatment for solid or hematological malignancy were included in the study. Patients admitted for less than 24 h in the ICU or patients who had stopped ICI treatment for more than 60 days before admission were not included. Investigational immunotherapies relying on inhibition of other checkpoints or mechanisms other than immune checkpoint inhibition and non-systemically administered immunotherapies were excluded.

All data were extracted from medical charts. Follow-up until 1 year after ICU admission was recorded.

Patients were then classified according to the reason for admission, whether related to an immune-related adverse event (irAE), an intercurrent adverse event not related to immunotherapy (intE) or a complication related to tumor progression (TumProg). Imputability of the ICI for irAEs was assessed by the physician in charge, discussed in multidisciplinary boards in most cases [27] and reviewed by investigators (AJ and VL), according to the World Health Organization Uppsala Monitoring Centre scale for standardized case causality assessment and organ-specific guidelines when available [16]. Tumor progression was defined as peri-tumoral hemorrhage, tumor obstruction or lymphangitis carcinomatosis. Intercurrent event was defined as any other medical condition neither related to tumor progression nor irAE. In case of concomitant tumor progression and immune-related or intercurrent event, the patient was classified a posteriori according to the reason for ICU admission as assessed by the physician in charge and reviewed by investigators (AJ and VL).

### Statistical analysis

Results were expressed as median and 25th and 75th quartiles [Q1–Q3] for quantitative data and numbers and percentages for categorical data. Quantitative variables were compared using the Wilcoxon test, and qualitative variables were compared using the Chi-square test with Yate’s continuity correction if needed. Baseline demographical, oncological, clinical and biological characteristics at ICU admission were described in the first table and relevant variables were tested for their association with irAE diagnosis (Fisher’s test) and 1-year mortality (logistic regression). A multivariate logistic regression model included variables that were significantly associated with 1-year mortality in univariate logistic regression and clinically relevant variables. Kaplan–Meier curves until 1 year after ICU admission were stratified using significant variables and compared using log-rank tests. Follow-up of patients after discharge from the ICU until death or end of follow-up was represented in a swimmer plot, where different colors represent different types of irAEs. Reintroduction of an ICI and complete responses according to iRECIST [28] were depicted by pictograms. All statistical tests were two-sided with an α level of 0.05. Statistics were managed using R software version 3.4.2 (R Foundation for Statistical Computing, Vienna, Austria; https://www.R-project.org/).

## Results

Between January 2013 and October 2019, 5644 cancer patients were admitted to the ICU and 112 of them (2%) were admitted within 60 days after an administration of ICI. Among them, 29 (26%) patients were admitted for an irAE, 44 (39%) patients were admitted for a complication related to tumor progression (TumProg) and 39 (35%) patients had another reason for ICU admission (intE) (Additional file 1: Figure S1).

**Fig. 1:**
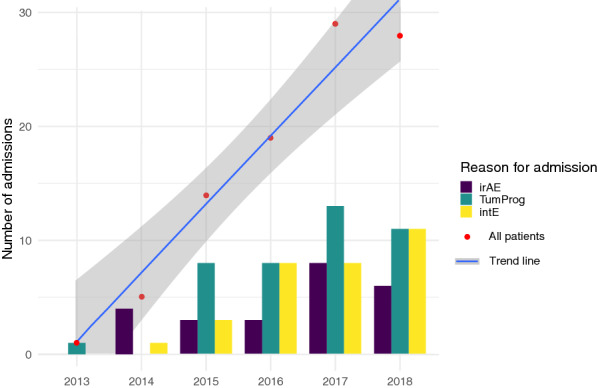
Bar plots of the number and reasons for admission over the study period (*p* < 0.001). *irAE* immune-related adverse event, *TumProg* complication related to tumor progression, *intE* intercurrent event

The absolute number of patients admitted within the course of an immunotherapy showed a significant increase from 2013 to 2018 (Mann–Kendall test *p* = 0.024), whereas the proportion of irAEs within these years remained stable (Chi-squared test for trend *p* = 0.298) (Fig. 1).

Altogether, patients admitted to the ICU within the course of an ICI treatment were mostly males (*n* = 68/112, 60.7%), had a median age of 64.7 [52.1–71.2] years and 61/112 (54.4%) had a performance status of 0 or 1. The most frequent malignancies were melanoma (*n* = 30/112, 26.8%), non-small cell lung cancer (NSCLC) (*n* = 32/112, 28.6%) and head and neck cancer (n = 15/112, 13.4%) (Additional file 2: Figure S2 panel B). Ninety-five patients (84.8%) had a metastatic disease at the time of admission.

Median time from ICI initiation to ICU admission was 51.5 [20.8–117.4] days, with a median 14 [7–27] days since last dose. Median Sequential Organ Failure Assessment (SOFA) score at admission was 4 [1–5].

Median ICU stay was 3 [1–5] days and 30/112 (26.8%) patients died in the ICU.

Fifteen patients (irAE (*n* = 5), intE (*n* = 7) and TumProg (*n* = 3), 13.4%) were lost to follow-up and 67/97 patients (69.1%) died before 1 year after ICU admission.

### Patients with irAE

Diagnoses of irAEs included pneumonitis (*n* = 8/29, 27.6%), colitis (*n* = 4/29, 13.8%), myocarditis (*n* = 3/29, 10.3%), metabolic disorder related to hypophysitis (*n* = 2/29, 6.9%) or diabetes mellitus (*n* = 3/29, 10.3%), nephritis (*n* = 2/29, 6.9%), hepatitis (*n* = 2/29, 6.9%), infusion-related hypersensitivity reaction (*n* = 2/29, 6.9%), pericarditis (*n* = 1/29, 3.4%), meningitis (*n* = 1/29, 3.4%) and encephalitis (*n* = 1/29, 3.4%) (Additional file 1: Figure S1).

All patients with pneumonitis, one patient with pericarditis and one patient with infusion-related hypersensitivity reaction were admitted for acute respiratory failures. The three patients with myocarditis, one patient with infusion-related hypersensitivity reaction and one patient with colitis were admitted for hemodynamic failure. The two patients with hepatitis were admitted for hepatic failure. All patients with diabetes and hypophysitis were admitted for hydroelectrolytic disorders. Meningitis and encephalitis patients were admitted for neurologic failure. Two patients with nephritis and two patients with colitis were admitted for acute kidney injury, whereas the last colitis patient was admitted for hemorrhage requiring emergency endoscopic evaluation.

The clinical characteristics and underlying disease of irAE patients at ICU admission are described in Table 1.

**Table 1:** Comparison of immune-related adverse events, intercurrent events and complications of tumor progression admissions

	IrAE (*n* = 29)	IntE (*n* = 39)	TumProg (*n* = 44)
Demographics and comorbidities			
Sex = M (*n*, %)	19 (66)	22 (56)	27 (61)
Age (median [IQR])	62.7 [52.9–71]	67.5 [61.9–74.1]	57.8 [45.5–67.8]
Comorbidities (*n*, %)			
Hypertension	11 (38)	25 (64)	13 (30)
Diabetes	4 (14)	6 (15)	3 (7)
Cardiac failure	3 (10)	5 (13)	4 (9)
COPD	1 (3)	6 (15)	4 (9)
Thromboembolic venous disease	5 (17)	7 (18)	7 (16)
Smoking status (*n*, %)			
Never	10 (34)	9 (23)	15 (34)
Past	6 (21)	10 (26)	9 (21)
Active	13 (45)	20 (51)	20 (46)
Weight loss (> 5 kg in the 3 preceding month) (*n*, %)	6 (21)	10 (26)	15 (34)
Performance status (*n*, %)			
≤ 2	23 (79)	31 (79)	30 (68)
> 2	2 (7)	8 (21)	9 (20)
BMI (kg/m^2^) (median [IQR])	24.8 [22.3–28.9]*	23.2 [21.1–27.8]	21.1 [19–24.7]*
Length of symptoms before admission (days) (median [IQR])	7 [3, 14]*	2 [0–4]**	2 [1–8.5]
Oncological characteristics			
Primary tumor site (n, %)			
Colorectal adenocarcinoma	0 (0)	1 (3)	1 (2)
Endometrial carcinoma	0 (0)	1 (3)	2 (5)
Prostate cancer	0 (0)	0 (0)	1 (2)
Ovarian cancer	1 (3)	2 (5)	0 (0)
Breast cancer	0 (0)	0 (0)	2 (5)
Cervical cancer	0 (0)	0 (0)	3 (7)
Squamous cell carcinoma	0 (0)	0 (0)	1 (2)
Esophagus cancer	0 (0)	0 (0)	1 (2)
Head and neck carcinoma	0 (0)	9 (23)	6 (14)
Urothelial carcinoma	5 (17)	7 (18)	4 (9)
Non-small cell lung cancer	7 (24)	12 (31)	13 (30)
Small cell lung cancer	0 (0)	0 (0)	1 (2)
Hodgkin lymphoma	1 (3)	0 (0)	0 (0)
Melanoma	14 (48)	7 (18)	9 (20)
Thymoma	1 (3)	0 (0)	0 (0)
State at admission (*n*, %)			
Localized	0 (0)	2 (5)	0 (0)
Locoregional	4 (14)	6 (15)	3 (7)
Metastatic	23 (79)	31 (79)	41 (93)
Number of metastatic sites (median [IQR])	2 [1, 3]	2 [1, 3]	2 [1, 3]
Length from diagnosis (months) (median [IQR])	13.5 [6.3–30.1]	16.6 [5.7–29.3]	16 [11.3–27.5]
Number of previous chemotherapeutic lines (median [IQR])	1 [0–1]	1 [0–2]	1 [1, 2]
Immune checkpoint inhibitors (*n*, %)			
Anti-PD-1			
NIVOLUMAB	9 (31)	19 (49)	22 (50)
PEMBROLIZUMAB	7 (24)	13 (33)	8 (18)
SPARTALIZUMAB	0 (0)	1 (3)	1 (2)
Anti-PDL-1			
ATEZOLIZUMAB	1 (3)	2 (5)	0 (0)
AVELUMAB	0 (0)	2 (5)	2 (5)
DURVALUMAB	1 (3)	1 (3)	4 (9)
Anti-CTLA4			
IPILIMUMAB	5 (17)*	1 (3)	2 (5)
TREMELIMUMAB	0 (0)	0 (0)	1 (2)
Anti-CTLA4/Anti-PD-1: IPILIMUMAB/NIVOLUMAB	6 (21*	0 (0)	4 (9)*
Length from ICI initiation and ICU admission (days) (median [IQR])	56 [30–84]	68 [20.5–199.5]	43.5 [19–88.5]
Length from last ICI dose (days) (median [IQR])	14 [17, 22]	14.5 [8.3–25.5]	15.5 [6.3–27.8]
Clinical and biological characteristics at ICU admission			
Blood pressure (systolic) (median [IQR])	129 [112.5–146]	124 [91–146.5]	123 [109.5–132.5]
Blood pressure (diastolic) (median [IQR])	72 [60–82.5]	69 [59–90.5]	71 [64.5–81]
Respiratory rate (median [IQR])	24 [19, 29]	21 [18–26.5]	22.5 [18, 25]
SpO_2_ (median (IQR)	97 [94.5–98]	96 [93–98.8]	97 [95–99]
Glasgow score for coma (median [IQR])	15 [14.8–15]	15 [13, 15]	15 [15–15]
SOFA Day 1 (median [IQR])	2 [1, 4]	4 [1, 6]	3 [3, 5]
Leucocytes (G/L) (median [IQR])	8.5 [6.5–16.1]	11.9 [7.4–18]	14.4 [8.7–19.2]
Neutrophils (G/L) (median [IQR])	5.3 [3.9–13.9]*	10.2 [6.1–16.2]*	12.3 [7–17.8]*
Lymphocytes (G/L) (median [IQR])	1 [0.8–1.6]	0.7 [0.5–1.6]	0.8 [0.4–1.4]
Neutrophil/Lymphocyte ratio (median [IQR])	6 [3.6–15.5]*	11.9 [5.5–23.3]	14.2 [6.2–22.4]*
Hemoglobin (g/dL) (median [IQR])	11.7 [10.3–13.2]*	11 [10.2–12]	9.9 [8.6–11.3]**
Platelets (G/L) (median [IQR])	305.5 [214.3–402.3]	271 [209.5–375.8]	288.5 [211.8–384]
Fibrinogen (g/L) (median [IQR])	4.7 [3.5–6]	5.1 [4.3–5.7]	5.5 [3.6–6.9]
Prothrombin Time (%) (median [IQR])	83 [74–91]*	74 [61–88]	70.5 [60–85]*
Creatinine (µM) (median [IQR])	106.5 [67–177.5]*	86 [59.3–148]	64 [54–97]*
Nitrogen (mM) (median [IQR])	9.1 [6.7–14.8]	8 [5.5–11.5]	7.7 [5.7–11.1]
Lactate (mM) (median [IQR])	1.7 [1.2–2.6]	2 [1.4–3.2]	2.4 [1.5–3.8]
pH (median [IQR])	7.4 [7.3–7.4]	7.4 [7.3–7.4]	7.4 [7.3–7.4]
Bilirubin (µM) (median [IQR])	8 [6, 12]	10 [7, 14]	10 [7–15.5]
Treatments in ICU and outcomes			
ICU length of stay (days) (median [IQR])	4 [1.3–8]	2 [1, 4]	3 [1.1–5.3]
Vasopressor therapy (n, %)	7 (24)	16 (41)	12 (27)
Mechanical ventilation (*n*, %)	6 (21)	12 (31)	13 (30)
Length of mechanical ventilation (days) (median [IQR])	8 [5.8–14.8]	2.5 [2–3.3]*	5 [2, 8]*
Non-invasive ventilation (*n*, %)	7 (24)	8 (21)	9 (21)
Renal replacement therapy (*n*, %)	3 (10)	1 (3)	2 (5)
CVVHF	1 (3)	0 (0)	1 (2)
Hemodialysis	2 (7)	1 (3)	1 (2)
Steroids (*n*, %)	18 (62)*	4 (10)**	17 (39)
ICU mortality (*n*, %)	5 (17)	9 (23)	16 (36)
Limitations during ICU stay (*n*, %)	4 (14) *	11 (28)	21 (48)*
Discontinuation of ICI (*n*, %)	18 (62)	22 (56)	30 (68)
One-year mortality (*n*, %)	10 (42)	21 (66)	36 (88)
	* = *p* < 0.05 ** = *p* < 0.001 against Others	* = *p* < 0.05 ** = *p* < 0.001 against irAE	* = *p* < 0.05 ** = *p* < 0.001 against irAE

*irAE* immune-related adverse event, *TumProg* complication related to tumor progression, *intE* intercurrent event, *COPD* chronic obstructive pulmonary disease, *ICI* immune checkpoint inhibitor, *ICU* intensive care unit, *CVVHF* continuous veno-venous hemofiltration, *SOFA* Sequential Organ Failure Assessment, *IQR* interquartile range

ICI at the time of admission included nivolumab (*n* = 9/29, 31%), pembrolizumab (*n* = 7/29, 24.1%), ipilimumab (*n* = 5/29, 17.2%) and the combination of ipilimumab and nivolumab in 6/29 (20.7%) patients (Additional file 2: Figure S2 panel A).

Immune-related adverse events were different between anti-CTLA4, anti-PD(L)-1 and combination therapy (*p* = 0.028), with pneumonitis being more common in patients treated with anti-PD(L)-1 (44% versus 0%, *p* = 0.03), whereas colitis was only diagnosed in patients treated with anti-CTLA4 or combination therapy (0 versus 36%, *p* = 0.027) (Additional file 2: Figure S2 panel C).

Vasopressor was required for 7/29 (24.1%) patients including 2/29 (6.9%) patients with extra-corporeal membrane oxygenation and 1/29 (3.4%) patient with renal replacement therapy. Oxygenation strategies included mechanical ventilation (*n* = 6, 20.7%), non-invasive ventilation only (*n* = 2/29, 6.9%) or high-flow nasal cannula (*n* = 2/29, 6.9%) or both non-invasive devices (*n* = 1/29, 3.4%). Renal replacement therapy without any other organ support was needed for 2/29 (6.9%) patients. No organ support was required for 13/29 (44.8%) patients admitted with metabolic disorder (*n* = 7/29, 24.1%) or for monitoring and observation (*n* = 6/29, 20.7%).

IrAEs were treated with steroids (*n* = 18/29, 62.1%) and second-line immunosuppression was required for 3/29 (10.3%) patients (immunoglobulins and plasma exchange (1/29, 3.4%) immunoglobulins, plasma exchange and tacrolimus (1/29, 3.4%) and cyclophosphamide (1/29, 3.4%)). All patients with diabetes, two patients with pneumonitis, and 6 patients with colitis (*n* = 1), hypophysitis (*n* = 1), myocarditis presenting with high-degree atrioventricular block (*n* = 1), meningitis (*n* = 1), pericarditis (*n* = 1) and hepatitis (*n* = 1) did not received steroids. Five out of 29 (17.2%) irAE patients died during ICU stay. One patient with hepatitis died 33 days after his first dose of ipilimumab for melanoma without any steroid treatment. One patient died from severe myocarditis 16 days after his first dose of nivolumab for thymic carcinoma despite treatment with steroids, immunoglobulins, and plasma exchange. Two NSCLC patients died from pneumonitis within 2 months of pembrolizumab and nivolumab therapy after treatment with steroids (*n* = 1) or steroids and cyclophosphamide (*n* = 1). Lastly, one NSCLC patient died from fulminant hepatitis 2.5 months after nivolumab therapy onset and despite steroid treatment.

After ICU discharge, 8/29 (27.6%) patients (diabetes (*n* = 2), hypophysitis (*n* = 1), meningitis (*n* = 1), infusion-related reaction (*n* = 1), colitis (*n* = 1), pneumonitis (*n* = 1), and pericarditis (*n* = 1)) were readministered the same ICI without any recurrence of significant adverse event (one grade 1 eosinophilia). Among these patients, 3/8 (37.5%) achieved complete responses, 2/8 (25%) patients had sustained partial responses, and tumor progression was diagnosed according to iRECIST for 3 (37.5%) of them.

Ten out of 24 (41.7%) irAE patients died before one year after ICU admission.

**Fig. 2:**
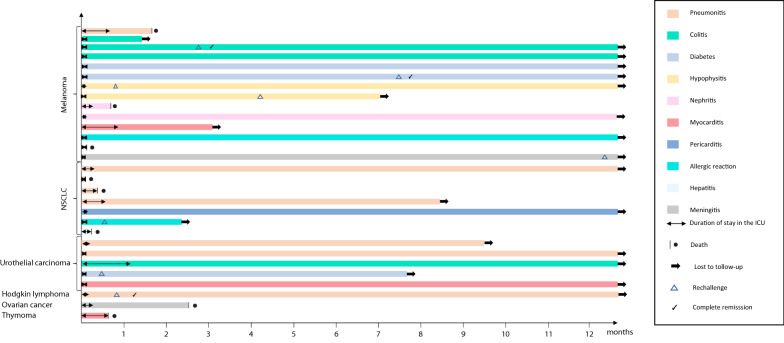
Swimmer plot of patients admitted for immune-related adverse events and evolution after discharge from ICU. An arrow represents the moment when the patient is lost to follow-up, a dot represents the moment of death. *NSCLC* non-small cell lung cancer, *ICU* intensive care unit

As described in the swimmer plot (Fig. 2), patients died within the ICU course (*n* = 5/29, 17.2%) or quickly after discharge (*n* = 3/29, 10.3%). No death was registered more than 3 months after ICU discharge and one lymphoma patient was still alive 5 years after an ICI-induced pneumonitis that required non-invasive ventilation in the ICU.

### Other reasons for ICU admission

For 83 other patients, the final diagnosis was not related to irAE. They are described in Table 1. Non-irAE diagnoses were mainly sepsis (*n* = 11/83, 13.3%), pneumonia (*n* = 10/83, 12%), hemorrhage (*n* = 9/83, 10.8%), cardiac failure (*n* = 7/83, 8.4%, including coronary event (*n* = 1), fluid overload and hypertension treated with diuretics only (*n* = 4), pericarditis with tumor cells on fluid examination (*n* = 1) and influenza-related myocarditis (*n* = 1)), peritonitis (*n* = 5/83, 6%), kidney failure (*n* = 4/83, 4.8%), pulmonary embolism (*n* = 2/83, 2.4%), and non-controlled tumor (*n* = 31/83, 37.3%).

Compared to other diagnoses, diagnosis of irAE was associated with melanoma (OR = 3.85 [1.42–10.66], *p* = 0.004) and a neutrophil/lymphocyte ratio lower than 10 (OR = 3.31 [1.16–10.19], *p* = 0.018), whereas non-irAE complications were associated with head and neck cancer (no irAE admission, *p* = 0.011). 

Durations of treatment before ICU admission were not different between the groups.

The proportion of patients who stopped ICI treatment after ICU discharge was not different between the groups (18/29 (62.1%), 22/39 (56.4%) and 28/44 (63.6%) for irAE, TumProg and intE, respectively, *p* = 0.942).

### Prognostic factors

Mortality rate in the ICU was, respectively, 17.2% (*n* = 5/29), 23.1% (*n* = 9/39) and 36.4% (*n* = 16/44) for ICU admission related to irAE, intE and TumProg (*p* = 0.159).

**Fig. 3:**
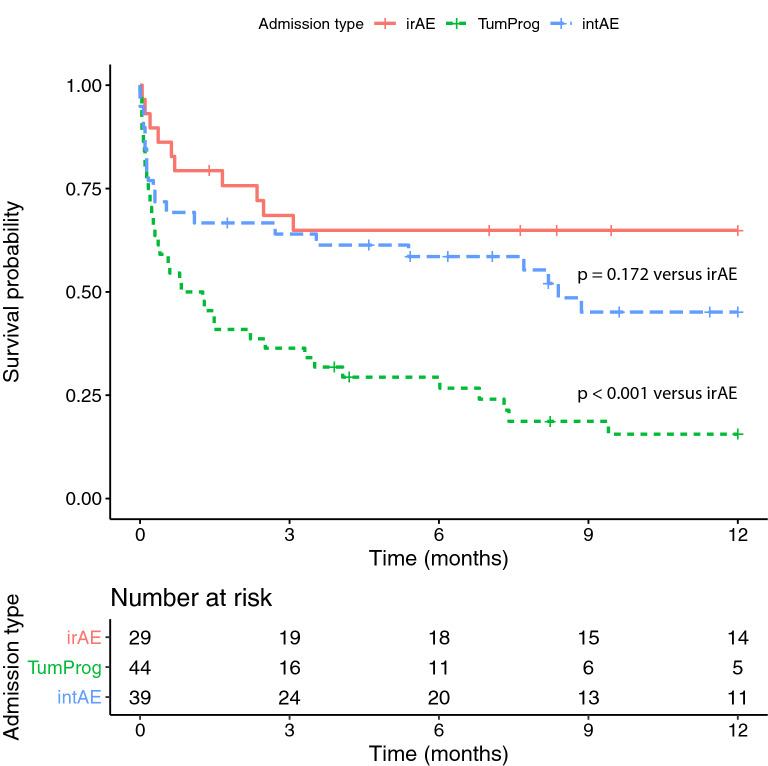
Kaplan–Meier curves for overall survival of patients admitted for irAE compared to intE (*p* = 0.172) and TumProg patients (*p* < 0.001) (irAE versus others *p* = 0.004). *irAE* immune-related adverse event, *TumProg* complication related to tumor progression, *intE* intercurrent event

Overall survival, censored at one year, was significantly higher for patients admitted with irAE compared to patients admitted for other reasons (*p* = 0.004). Specifically, survival for irAE patients was significantly better compared with TumProg patients (*p* < 0.001) and not significantly different compared with intE patients (*p* = 0.172) (Fig. 3).

Factors associated with one-year mortality in multivariate analysis (Table 2) were related to tumor site (NSCLC versus others (OR = 1.28 [1.07–1.52], *p* = 0.007)), duration of ICI treatment before ICU admission (OR = 0.973 [0.952–0.994], *p* = 0.014)), reason for ICU admission (ICU admission related to a complication of tumor progression (OR = 1.35 [1.14–1.59], *p* < 0.001)) and SOFA score at ICU admission (OR = 1.05 [1.03–1.08], *p* < 0.001).

**Table 2:** Factors associated with 12-month mortality in univariate and multivariate analysis in patients admitted in the intensive care unit (ICU) within 60 days after last dose of immune checkpoint inhibitor (ICI)

	OR for mortality (univariate logistic regression model)	OR for mortality (multivariate logistic regression model)
Reason for admission		
Immune-related adverse event	Ref	
Intercurrent complication	1.27 [0.98–1.65]	
Complication of tumor progression	1.59 [1.29–1.94]	1.35 [1.14–1.59]
Demographics and comorbidities		
Weight loss (> 5 kg in the 3 preceding months)	1.24 [1.00–1.52]	
Performance status > 2	1.27 [1.01–1.60]	1.15 [0.94–1.40]
Oncological characteristics		
Primary tumor site		
Non-small cell lung cancer	1.24 [1.02–1.51]	1.28 [1.07–1.52]
Melanoma	0.66 [0.54–0.78]	
Metastatic disease at admission	1.46 [1.10–1.93]	
Length from ICI initiation (month)	0.974 [0.951–0.997]	0.973 [0.952–0.994]
Clinical and biological characteristics at ICU admission		
SOFA Day 1	1.04 [1.01–1.07]	1.05 [1.03–1.08]
Neutrophil–lymphocyte ratio > 10	1.33 [1.09–1.62]	
Treatments in ICU		
Mechanical ventilation	1.19 [0.97–1.46]	
Non-invasive ventilation	1.25 [1.01–1.55]	
Renal replacement therapy	1.16 [0.79–1.71]	
Steroids	1.12 [0.92–1.36]	

Hazard ratios and their 95% confidence interval are shown for factors associated with 12-month mortality in univariate and multivariate analyses

Additional file 3 shows survival curves according to ICI molecule (panel A) and tumor site (panel B).

## Discussion

This study describes patients admitted to the ICU after ICI treatment. The main result of our study is the better outcome for irAE patients compared with other reasons of ICU admission. Median survival exceeded one year for patients with irAE.

Immune checkpoint inhibitors have reshaped the standard of care in oncology wards and are increasingly encountered in intensive care units, as shown by the exponential increase of the number of admissions in our study. Intensivists need to be aware of these new treatments leading to high survival rates with a good quality of life, even in patients with advanced cancer [1]. These treatments, leading to an increased immune response, have been associated with several immune-related disorders [29], most of which remain low grade and can be easily managed. However, toxicity-related mortality under ICI can reach 0.36% for anti-PD1 and 1.23% for anti-PD1/anti-CTLA4 combination [30]. With an increased number of treated patients, number of fatal irAEs reported has increased more than threefold between 2015 and 2017 [30]. Therefore, intensivists should be aware of such severe complications with potential good outcomes after early diagnosis and treatment. Indeed, other complications may be easily ruled out with a simple diagnostic strategy relying on a close collaboration between oncologists and intensivists. Then immunosuppressive treatment including steroids should be quickly prescribed to reverse irAE [20, 31, 32] Systemic steroids are recommended for grade 3 and 4 immune-related adverse events [20, 21, 32, 33]. For steroid-refractory irAEs, a personalized management based on the predominant immune infiltrate is advised [22, 23]. However, there are not yet recommendations for salvage therapy when steroids fail to decrease toxicity. Thus, a close collaboration between oncologists and intensivists must be of importance. Based on treatment approaches for primary autoimmune disorders, early treatment by anti-TNFα in colitis [24, 34] and plasma exchange in neurological toxicities [25] have been advocated.

The second result of importance of the study is the kind of admitted patients. Although ICI are usually prescribed for metastatic disease within first- or second-line treatment, patients may keep high performance status. Such patients could benefit from ICU admission. Moreover, in this study as in previous ones, ICI could be safely readministered and could lead to long-term responses [35, 36]. Also, some patients with highly severe AE and in whom ICI was stopped have shown long-term response or stability. Immune-related adverse events have been shown to correlate with response rate, and some studies describe a better prognosis in patients experiencing irAEs [37–39]. Therefore, such adverse events do not lead to palliative treatment. However, performance status should be evaluated before ICU admission as it has been consistently associated with mortality in intensive care cancer patients [40].

Also, this study describes the clinical heterogeneity of such patients in the context of intensive care; the 3 most frequent diagnoses (pneumonitis, colitis and cardiac disorders) representing only approximately half of the patients. These adverse events have been already described [2]. Particularly, colitis has been associated with anti-CTLA4, whereas pneumonitis has been related to anti-PD1 treatment [41], as shown in our study. Moreover, we confirm the risk of severe AE when ICI combinations are used [2, 30, 42]. If our small sample size does not allow us to compare mortality across the different types of irAEs, larger cohorts from outside of the ICU have described high mortality rates [30] in myocarditis [16–19], pneumonitis [4–6] and hepatitis [11–13] patients. Although rarely lethal, colitis has been reported as the first cause of treatment-related death in patients under anti-CTLA4 therapy [30].

Some irAEs, such as diabetic ketoacidosis or acute hypophysitis, carry a good prognosis compared with more frequent conditions leading to ICU admissions in oncological patients. Such diagnoses should be easily raised and managed by intensivists even in patients with advanced cancer. Moreover, such irAEs can be managed without immunosuppressive treatment [36]. In our cohort, patients admitted for diabetes ketoacidosis and several other immune-related events did not receive steroids, highlighting the need for raising awareness of this kind of complication and establishing multidisciplinary protocols for the treatment of these patients, especially the ones who are critically ill.

There are several limitations in this study. First, this was a retrospective study including only patients admitted to the ICU. Although irAEs are rare, this study could include 112 patients treated with ICI and 29 irAEs. Compared to case reports, this is the largest study describing irAEs in the ICU setting. Moreover, comparison with other reasons of ICU admission in patients receiving ICI could be performed.

In such retrospective studies, admission bias remains the most important limitation. Indeed, most patients in our study had a high performance status that may not reflect all cancer patients, preventing a direct comparison to patients treated with conventional chemotherapies admitted in the ICU. However patients admitted to the ICU for non-immune-related reasons while receiving a treatment by an ICI have been compared to patients admitted for irAEs.

Third, we chose a timeframe of 60 days from the last dose to admission in the ICU. If time from first dose to onset of irAE has been deeply studied and varies largely among irAEs [2], time from last dose to irAE has been seldom reported. On the one hand, some irAEs have been reported up to 2 years after ICI cessation [43]. On the other hand, time from last dose to irAE does not seem to vary much between early and late-occurring irAEs, with a median time of 2 weeks in both colitis [44] and nephritis [45], similar to our findings. Thus, our 2-month timeframe between last ICI dose and admission in the ICU unlikely resulted in a significant admission bias, whereas it prevented issues concerning imputability of the ICI or comparability between the irAE group and patients admitted for non-immune-related reasons long after cessation of ICI.

Lastly, irAEs were heterogeneous and no risk factor could be determined for each type of irAE. However, the aim of our study was to increase knowledge of this kind of adverse events for intensivists. In the future, with the increasing number of ICI-treated patients, new combinations and probably higher risk of ICU admission, adverse events will be better evaluated [46].

## Conclusions

In conclusion, intensivists should be aware of irAEs even in patients with advanced cancer. Such adverse events, with early diagnosis and treatment, may be associated with good outcomes even in the case of severe organ failure and in metastatic settings. These results justify ICU admission for such patients, but a close collaboration between oncologists and intensivists for diagnostic procedure and immunosuppressive treatment remains essential.

## Supplementary information

**Additional file 1: Figure S1.** Eligibility and classification of patients admitted to the ICU over the study period. ICI: Immune checkpoint inhibitor.

**Additional file 2: Figure S2.** A: Pie chart of ICI treatments (*n* = 112). B: Pie chart of primary tumor sites (*n* = 112). C: Radar chart of types of irAE according to class of ICI (*n* = 29). Each class of ICI is represented in a different color and the number of each type of complication is featured on an axis. D: Status at ICU discharge according to the type of irAE (*n* = 29). The number patients alive and dead at discharge from ICU for each type of complication is featured on axis in blue and yellow color.

**Additional file 3: Figure S3**. Kaplan–Meier curves for overall survival stratified for class of immune checkpoint inhibitor (*p* = 0.19) and tumor type (*p* < 0.001).

## Data Availability

The datasets used and analyzed during the current study are available from the corresponding author on reasonable request.

## Abbreviations

CTLA-4: Cytotoxic T-lymphocyte antigen 4
ICI: Immune checkpoint inhibitors
ICU: Intensive care unit
IntE: Intercurrent event
IRAE: Immune-related adverse events
NSCLC: Non-small cell lung cancer
PD-1: Programmed cell death 1
PDL-1: Programmed cell death ligand 1
SOFA: Sequential Organ Failure Assessment
TumProg: Tumor progression

## References

[1] Hirsch L, Zitvogel L, Eggermont A, Marabelle A (2019). PD-Loma: a cancer entity with a shared sensitivity to the PD-1/PD-L1 pathway blockade. Br J Cancer.

[2] Martins F, Sofiya L, Sykiotis GP, Lamine F, Maillard M, Fraga M (2019). Adverse effects of immune-checkpoint inhibitors: epidemiology, management and surveillance. Nat Rev Clin Oncol.

[3] Geraud A, Gougis P, Vozy A, Anquetil C, Allenbach Y, Romano E, et al. Clinical pharmacology and interplay of immune checkpoint agents: a Yin-Yang balance. Annu Rev Pharmacol Toxicol. 2020. 10.1146/annurev-pharmtox-022820-093805 32871087

[4] Khunger M, Rakshit S, Pasupuleti V, Hernandez AV, Mazzone P, Stevenson J (2017). Incidence of pneumonitis with use of programmed death 1 and programmed death-ligand 1 inhibitors in non-small cell lung cancer. Chest.

[5] Naidoo J, Wang X, Woo KM, Iyriboz T, Halpenny D, Cunningham J (2017). Pneumonitis in patients treated with anti-programmed death-1/programmed death ligand 1 therapy. J Clin Oncol.

[6] Sears CR, Peikert T, Possick JD, Naidoo J, Nishino M, Patel SP (2019). Knowledge gaps and research priorities in immune checkpoint inhibitor-related pneumonitis. An Official American Thoracic Society Research Statement. Am J Respir Crit Care Med..

[7] Kao JC, Liao B, Markovic SN, Klein CJ, Naddaf E, Staff NP (2017). neurological complications associated with anti-programmed death 1 (PD-1) antibodies. JAMA Neurol.

[8] Graus F, Dalmau J (2019). Paraneoplastic neurological syndromes in the era of immune-checkpoint inhibitors. Nat Rev Clin Oncol.

[9] Moreira A, Loquai C, Pföhler C, Kähler KC, Knauss S, Heppt MV (1990). Myositis and neuromuscular side-effects induced by immune checkpoint inhibitors. Eur J Cancer Oxf Engl (1990).

[10] Allenbach Y, Anquetil C, Manouchehri A, Benveniste O, Lambotte O, Lebrun-Vignes B (2020). Immune checkpoint inhibitor-induced myositis, the earliest and most lethal complication among rheumatic and musculoskeletal toxicities. Autoimmun Rev.

[11] Suzman DL, Pelosof L, Rosenberg A, Avigan MI (2018). Hepatotoxicity of immune checkpoint inhibitors: An evolving picture of risk associated with a vital class of immunotherapy agents. Liver Int.

[12] Riveiro-Barciela M, Barreira-Díaz A, Vidal-González J, Muñoz-Couselo E, Martínez-Valle F, Viladomiu L (2020). Immune-related hepatitis related to checkpoint inhibitors: clinical and prognostic factors. Liver Int.

[13] Peeraphatdit (Bee) T, Wang J, Odenwald MA, Hu S, Hart J, Charlton MR (2020). Hepatotoxicity from immune checkpoint inhibitors: a systematic review and management recommendation. Hepatology.

[14] Varricchi G, Galdiero MR, Tocchetti CG (2017). Cardiac toxicity of immune checkpoint inhibitors: cardio-oncology meets immunology. Circulation.

[15] Ball S, Ghosh RK, Wongsaengsak S, Bandyopadhyay D, Ghosh GC, Aronow WS (2019). Cardiovascular toxicities of immune checkpoint inhibitors. J Am Coll Cardiol.

[16] Bonaca MP, Olenchock BA, Salem J-E, Wiviott SD, Ederhy S, Cohen A (2019). Myocarditis in the setting of cancer therapeutics: proposed case definitions for emerging clinical syndromes in cardio-oncology. Circulation.

[17] Moslehi JJ, Salem J-E, Sosman JA, Lebrun-Vignes B, Johnson DB (2018). Increased reporting of fatal immune checkpoint inhibitor-associated myocarditis. Lancet Lond Engl.

[18] Mahmood SS, Fradley MG, Cohen JV, Nohria A, Reynolds KL, Heinzerling LM (2018). Myocarditis in patients treated with immune checkpoint inhibitors. J Am Coll Cardiol.

[19] Johnson DB, Balko JM, Compton ML, Chalkias S, Gorham J, Xu Y (2016). Fulminant myocarditis with combination immune checkpoint blockade. N Engl J Med.

[20] Brahmer JR, Lacchetti C, Schneider BJ, Atkins MB, Brassil KJ, Caterino JM (2018). Management of immune-related adverse events in patients treated with immune checkpoint inhibitor therapy: American Society of Clinical Oncology Clinical Practice Guideline. J Clin Oncol.

[21] Puzanov I, Diab A, Abdallah K, Bingham CO, Brogdon C, on behalf of the Society for Immunotherapy of Cancer Toxicity Management Working Group (2017). Managing toxicities associated with immune checkpoint inhibitors: consensus recommendations from the Society for Immunotherapy of Cancer (SITC) Toxicity Management Working Group. J Immunother Cancer..

[22] Martins F, Sykiotis GP, Maillard M, Fraga M, Ribi C, Kuntzer T (2019). New therapeutic perspectives to manage refractory immune checkpoint-related toxicities. Lancet Oncol.

[23] Esfahani K, Elkrief A, Calabrese C, Lapointe R, Hudson M, Routy B, et al. Moving towards personalized treatments of immune-related adverse events. Nat Rev Clin Oncol. 2020;1–12. 10.1038/s41571-020-0352-8 32246128

[24] Johnson DH, Zobniw CM, Trinh VA, Ma J, Bassett RL, Abdel-Wahab N (2018). Infliximab associated with faster symptom resolution compared with corticosteroids alone for the management of immune-related enterocolitis. J Immunother Cancer.

[25] Safa H, Johnson DH, Trinh VA, Rodgers TE, Lin H, Suarez-Almazor ME (2019). Immune checkpoint inhibitor related myasthenia gravis: single center experience and systematic review of the literature. J Immunother Cancer.

[26] Taccone FS, Artigas AA, Sprung CL, Moreno R, Sakr Y, Vincent J-L (2009). Characteristics and outcomes of cancer patients in European ICUs. Crit Care Lond Engl.

[27] Michot J-M, Lappara A, Le Pavec J, Simonaggio A, Collins M, De Martin E (1990). The 2016–2019 ImmunoTOX assessment board report of collaborative management of immune-related adverse events, an observational clinical study. Eur J Cancer Oxf Engl.

[28] Seymour L, Bogaerts J, Perrone A, Ford R, Schwartz LH, Mandrekar S (2017). iRECIST: guidelines for response criteria for use in trials testing immunotherapeutics. Lancet Oncol.

[29] Xu C, Chen Y-P, Du X-J, Liu J-Q, Huang C-L, Chen L (2018). Comparative safety of immune checkpoint inhibitors in cancer: systematic review and network meta-analysis. BMJ.

[30] Wang DY, Salem J-E, Cohen JV, Chandra S, Menzer C, Ye F (2018). Fatal toxic effects associated with immune checkpoint inhibitors: a systematic review and meta-analysis. JAMA Oncol.

[31] Lemiale V, Meert A-P, Vincent F, Darmon M, Bauer PR, Groupe de Recherche en Reanimation Respiratoire du patient d’Onco-Hématologie (Grrr-OH) (2019). Severe toxicity from checkpoint protein inhibitors: What intensive care physicians need to know?. Ann Intensive Care..

[32] Thompson JA, Schneider BJ, Brahmer J, Andrews S, Armand P, Bhatia S (2019). Management of immunotherapy-related toxicities, version 1.2019. J Natl Compr Cancer Netw JNCCN..

[33] Champiat S, Lambotte O, Barreau E, Belkhir R, Berdelou A, Carbonnel F (2016). Management of immune checkpoint blockade dysimmune toxicities: a collaborative position paper. Ann Oncol.

[34] Perez-Ruiz E, Minute L, Otano I, Alvarez M, Ochoa MC, Belsue V (2019). Prophylactic TNF blockade uncouples efficacy and toxicity in dual CTLA-4 and PD-1 immunotherapy. Nature.

[35] Simonaggio A, Michot JM, Voisin AL, Pavec JL, Collins M, Lallart A (2019). Evaluation of readministration of immune checkpoint inhibitors after immune-related adverse events in patients with cancer. JAMA Oncol.

[36] Cuzzubbo S, Tetu P, Guegan S, Ursu R, Belin C, Villaros LS (2020). Reintroduction of immune-checkpoint inhibitors after immune-related meningitis: a case series of melanoma patients. J Immunother Cancer.

[37] Weber JS, Hodi FS, Wolchok JD, Topalian SL, Schadendorf D, Larkin J (2017). Safety profile of nivolumab monotherapy: a pooled analysis of patients with advanced melanoma. J Clin Oncol Off J Am Soc Clin Oncol.

[38] Haratani K, Hayashi H, Chiba Y, Kudo K, Yonesaka K, Kato R (2018). Association of immune-related adverse events with nivolumab efficacy in non–small-cell lung cancer. JAMA Oncol.

[39] Das S, Johnson DB (2019). Immune-related adverse events and anti-tumor efficacy of immune checkpoint inhibitors. J Immunother Cancer.

[40] Azevedo LCP, Caruso P, Silva UVA, Torelly AP, Silva E, Rezende E (2014). Outcomes for patients with cancer admitted to the ICU requiring ventilatory support: results from a prospective multicenter study. Chest.

[41] Khoja L, Day D, Wei-Wu Chen T, Siu LL, Hansen AR (2017). Tumour- and class-specific patterns of immune-related adverse events of immune checkpoint inhibitors: a systematic review. Ann Oncol.

[42] Chan KK, Bass AR. Autoimmune complications of immunotherapy: pathophysiology and management. BMJ. 2020. 369. https://www.bmj.com/content/369/bmj.m736. Accessed 23 July 2020. 10.1136/bmj.m736 32253223

[43] Couey MA, Bell RB, Patel AA, Romba MC, Crittenden MR, Curti BD (2019). Delayed immune-related events (DIRE) after discontinuation of immunotherapy: diagnostic hazard of autoimmunity at a distance. J Immunother Cancer.

[44] Soularue E, Lepage P, Colombel JF, Coutzac C, Faleck D, Marthey L (2018). Enterocolitis due to immune checkpoint inhibitors: a systematic review. Gut.

[45] Cortazar FB, Kibbelaar ZA, Glezerman IG, Abudayyeh A, Mamlouk O, Motwani SS (2020). Clinical features and outcomes of immune checkpoint inhibitor-associated AKI: a multicenter study. J Am Soc Nephrol JASN.

[46] Tang J, Yu JX, Hubbard-Lucey VM, Neftelinov ST, Hodge JP, Lin Y (2018). The clinical trial landscape for PD1/PDL1 immune checkpoint inhibitors. Nat Rev Drug Discov.

