# Supplementary figures and images for: Immune-related adverse events: a retrospective look into the future of oncology in the intensive care unit
Source: Ann Intensive Care. 2020 Oct 16;10:143. doi: 10.1186/s13613-020-00761-w (PMC7567777; doi:10.1186/s13613-020-00761-w)

**A**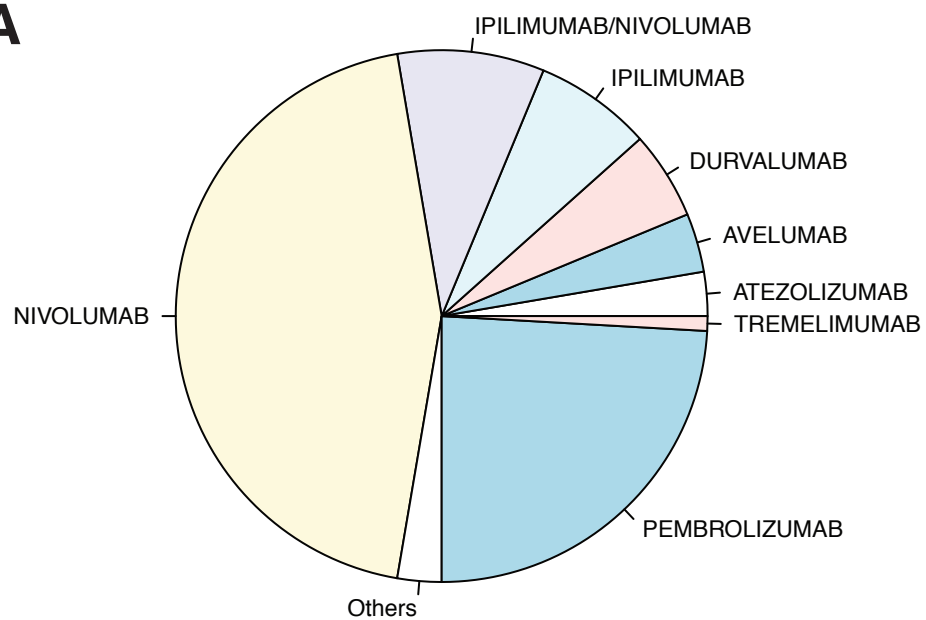**B**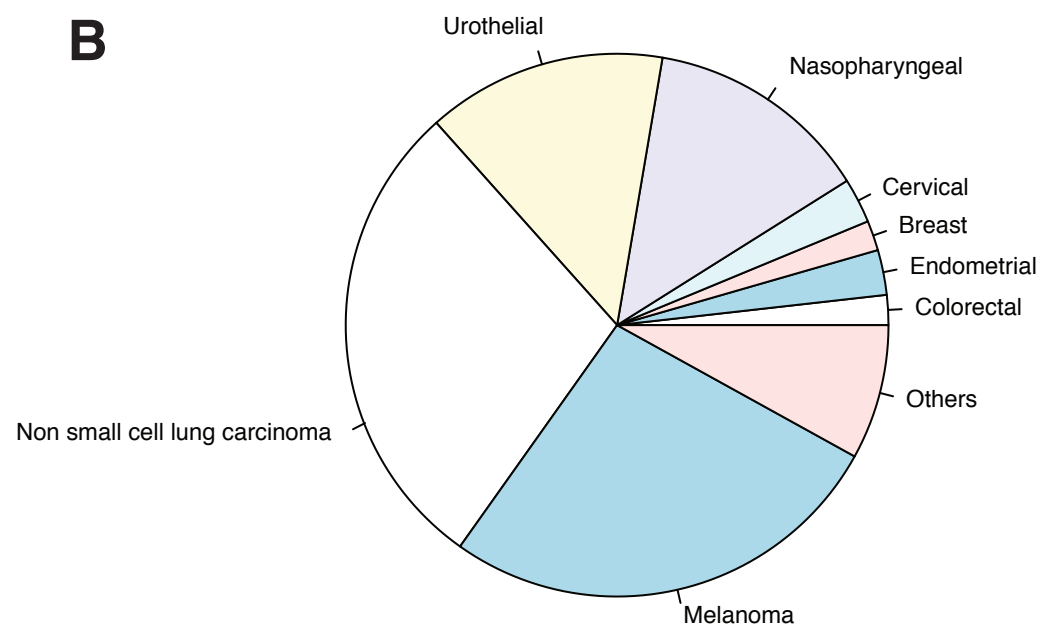**C**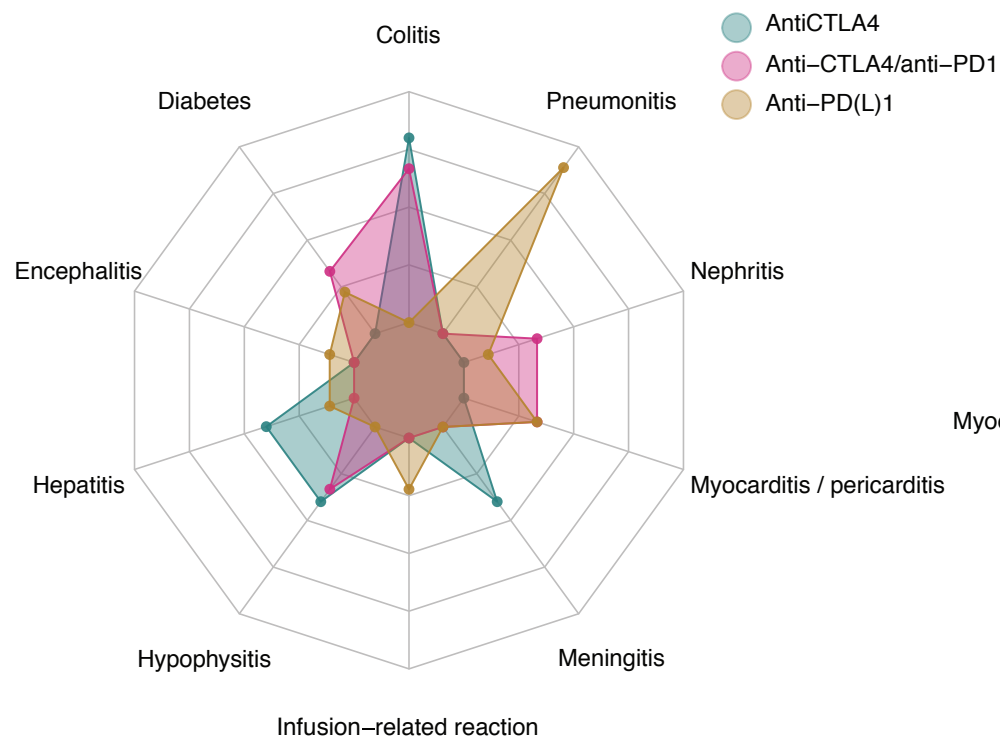**D**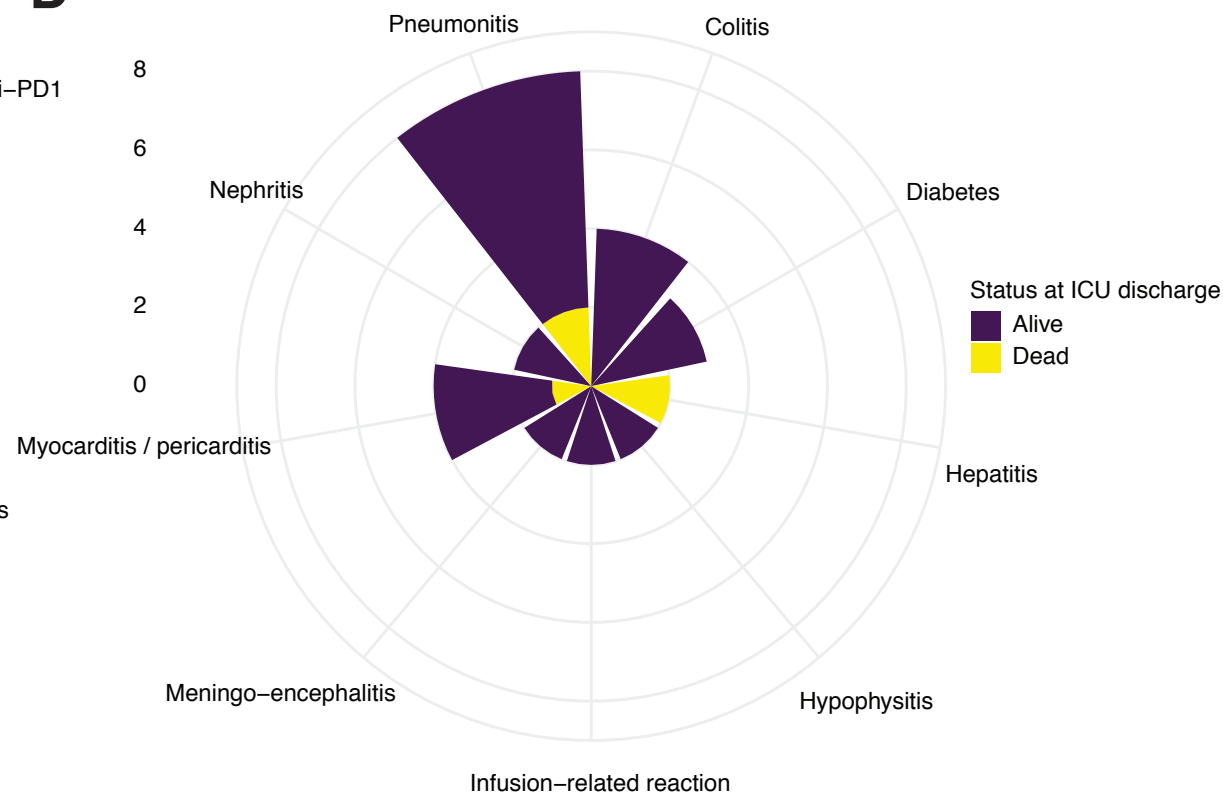

Supplement: Supplementary file 1 — Additional file 1: Figure S1. Eligibility and classification of patients admitted to the ICU over the study period. ICI: Immune checkpoint inhibitor. [file 13613_2020_761_MOESM1_ESM.pdf]

A

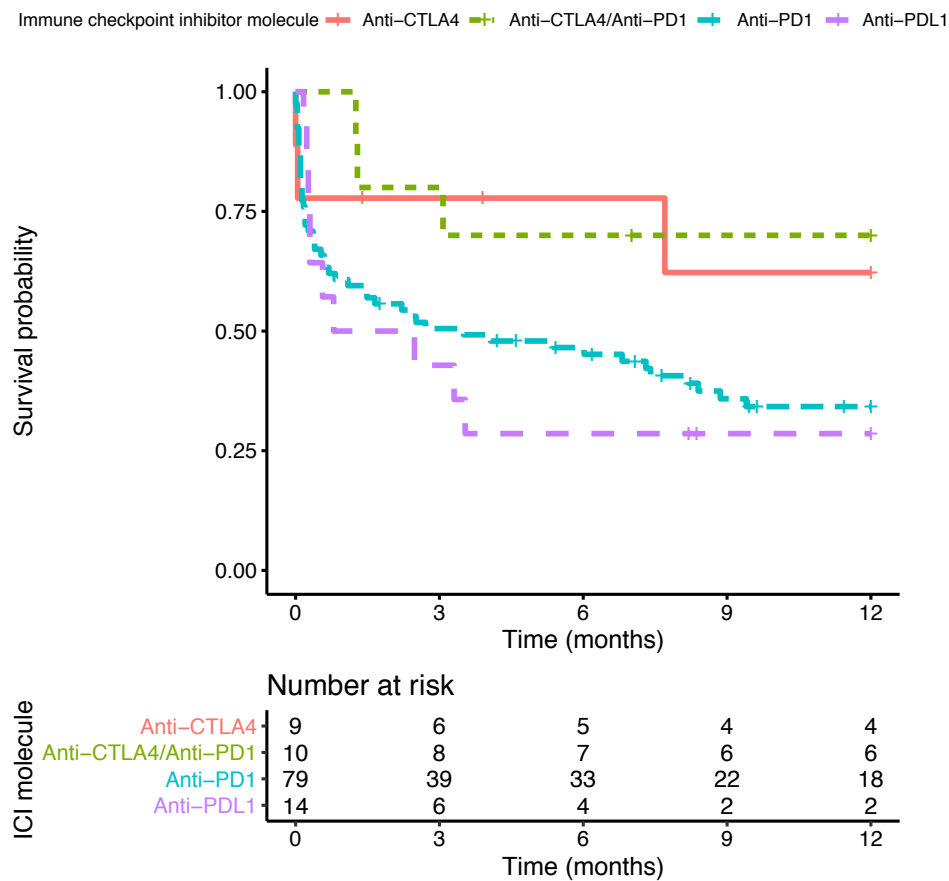

B

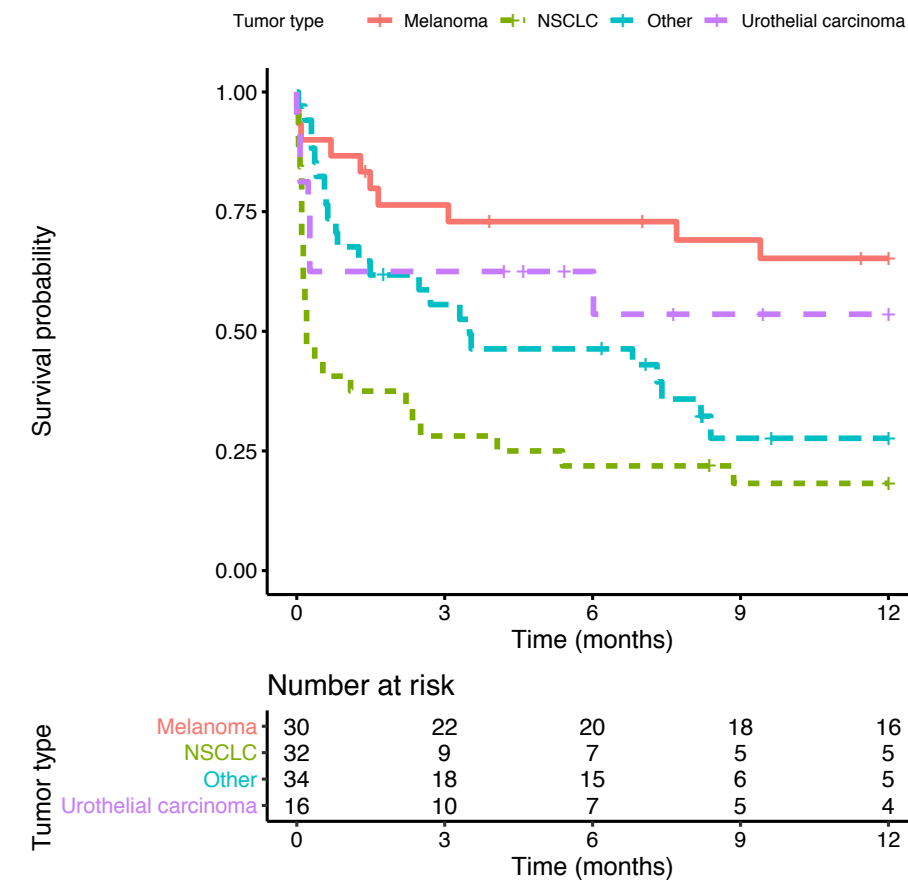

Supplement: Supplementary file 2 — Additional file 2: Figure S2. A: Pie chart of ICI treatments (n = 112). B: Pie chart of primary tumor sites (n = 112). C: Radar chart of types of irAE according to class of ICI (n = 29). Each class of ICI is represented in a different color and the number of each type of complication is featured on an axis. D: Status at ICU discharge according to the type of irAE (n = 29). The number patients alive and dead at discharge from ICU for each type of complication is featured on axis in blue and yellow color. [file 13613_2020_761_MOESM2_ESM.pdf]

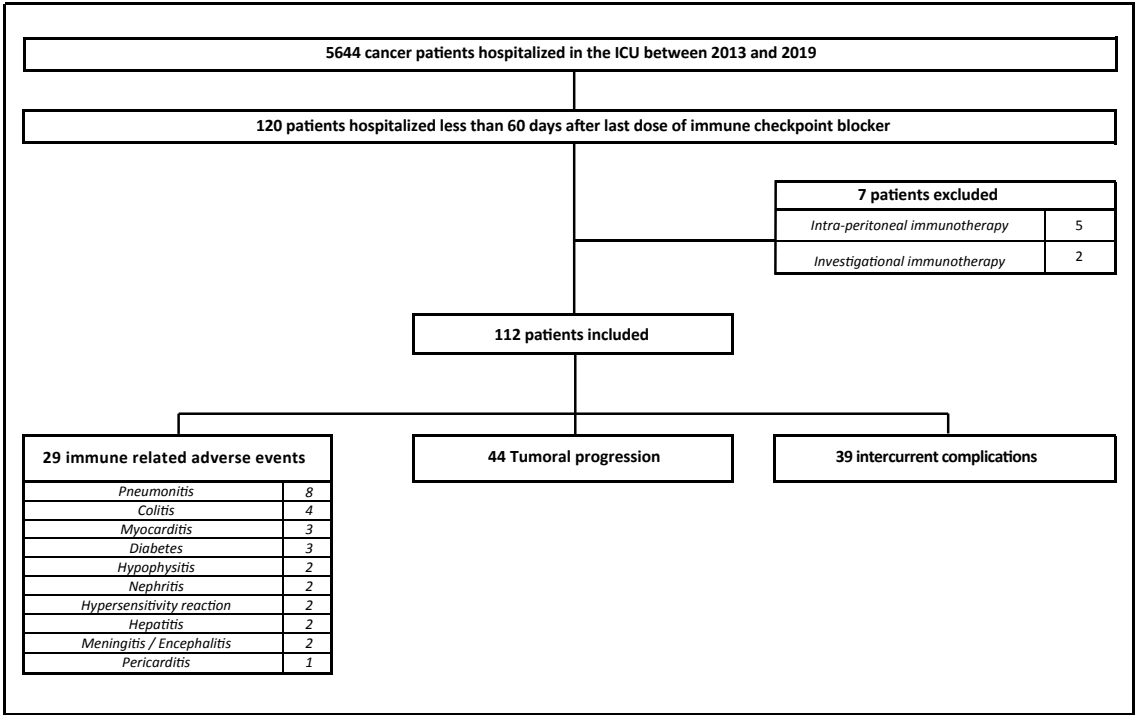

Supplement: Supplementary file 3 — Additional file 3: Figure S3. Kaplan–Meier curves for overall survival stratified for class of immune checkpoint inhibitor (p = 0.19) and tumor type (p < 0.001). [file 13613_2020_761_MOESM3_ESM.pdf]
